# αβ T cell receptors as predictors of health and disease

**DOI:** 10.1038/cmi.2014.134

**Published:** 2015-01-26

**Authors:** Meriem Attaf, Eric Huseby, Andrew K Sewell

**Affiliations:** 1 Cardiff University School of Medicine, Cardiff, UK; 2 Department of Pathology, University of Massachusetts Medical School, Worcester, MA, USA

**Keywords:** T cell receptor (TCR), TCR repertoire, TCR diversity, TCR clonotype, TCR bias, Deep sequencing

## Abstract

The diversity of antigen receptors and the specificity it underlies are the hallmarks of the cellular arm of the adaptive immune system. T and B lymphocytes are indeed truly unique in their ability to generate receptors capable of recognizing virtually any pathogen. It has been known for several decades that T lymphocytes recognize short peptides derived from degraded proteins presented by major histocompatibility complex (MHC) molecules at the cell surface. Interaction between peptide-MHC (pMHC) and the T cell receptor (TCR) is central to both thymic selection and peripheral antigen recognition. It is widely assumed that TCR diversity is required, or at least highly desirable, to provide sufficient immune coverage. However, a number of immune responses are associated with the selection of predictable, narrow, or skewed repertoires and public TCR chains. Here, we summarize the current knowledge on the formation of the TCR repertoire and its maintenance in health and disease. We also outline the various molecular mechanisms that govern the composition of the pre-selection, naive and antigen-specific TCR repertoires. Finally, we suggest that with the development of high-throughput sequencing, common TCR ‘signatures' raised against specific antigens could provide important diagnostic biomarkers and surrogate predictors of disease onset, progression and outcome.

## Introduction

T cells orchestrate immune responses by interrogating protein expression *via* peptides cradled in major histocompatibility complex (MHC) molecules at the cell surface. The T cell receptor (TCR) is the fundamental unit underlying all peptide-MHC (pMHC) recognition events. In the thymus, T cell signalling induced by self-pMHC engagement contributes to the process of selection at the double-positive stage, whereby only a fraction of thymocytes bearing TCRs within a narrow affinity range are permitted to differentiate into mature T cells.^1^ In secondary lymphoid organs, ligation of the TCR to pMHC provides the cell with the earliest signals required for the execution of a complex differentiation programme associated with effector function. In the steady state, homeostasis of T cell numbers is also MHC-dependent.^2^

**Figure 1:**
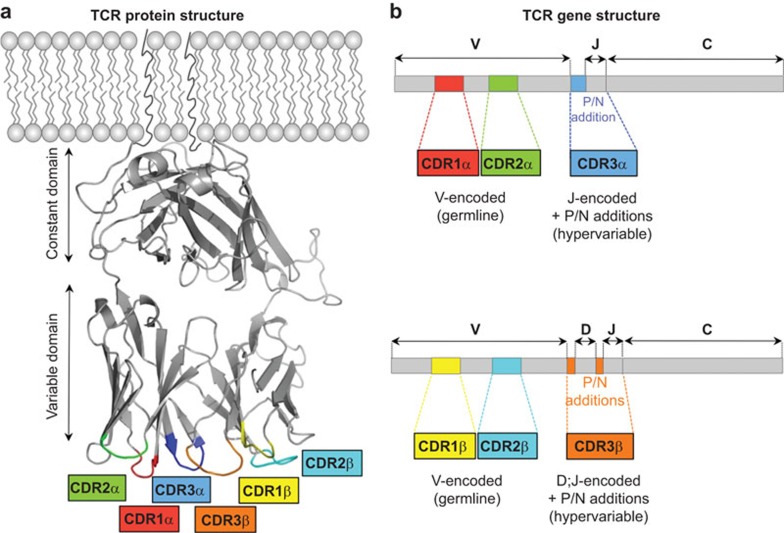
TCR protein and gene structure. (**a**) Structure of the AS01 TCR.^39^ The TCR-α and TCR-β CDR loops are short hairpin turns linking adjacent β-strands. (**b**) CDR1 and CDR2 are entirely encoded in the germline V genes, whereas CDR3 lies at the junction between the rearranged V and J segments (TCR-α) and V, D and J segments (TCR-β). The CDR3 junctional sites are assembled by random addition and deletion of template and non-template nucleotides (blue for TCR-α and orange for TCR-β). CDR, complementarity-determining region; TCR, T cell receptor.

**Figure 2:**
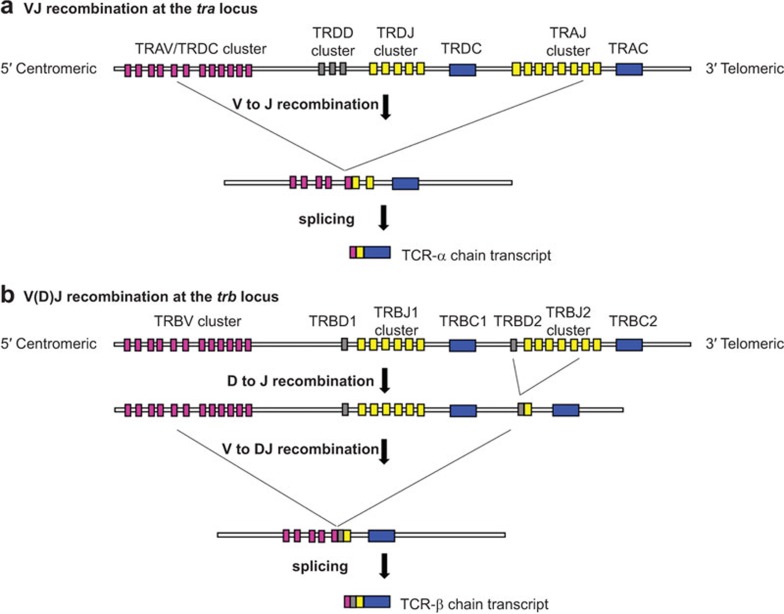
Gene rearrangement at the *tr* loci. (**a**) VJ recombination at the *tra* locus. The *tra* locus (in which the TCR-δ, or *trd* locus is also embedded) comprises a 5′ V gene segment cluster (46 TRAV segments) followed by a central J cluster (51 TRAJ segments) and a single C gene segment (TRAC). TCR-δ D and J segments (TRDD and TRDJ, respectively) are also present in the locus. V to J recombination brings together one of many TRAV segments to one of many TRAJ segments. The intervening sequences are spliced out, producing a TCR-α transcript in which V, J and C segments are directly adjacent. (**b**) VDJ recombination at the *trb* locus. The *trb* locus is composed of a 5′ V cluster (48 TRBV gene segments) followed by two 3′ TRBD–TRBJ–TRBC clusters. VDJ recombination is a two-step, ordered process. D to J recombination occurs first, juxtaposing TRBD1 to one of the six TRBJ1 segments or TRBD2 to one the seven TRBJ2 segments. V to DJ recombination subsequently brings the rearranged DJ join to one of many TRBV segments. The intervening sequences are then spliced out, generating a TCR-β transcript in which, V, D, J and C segments are directly adjacent. TCR, T cell receptor.

The TCR is a heterodimer of one α and one β chain, or one γ and one δ chain, which are disulphide-linked.^3,4^ Each TCR chain is composed of a constant and a variable domain, followed by a membrane-spanning region and a short cytosolic tail. Diversity in the TCR is predominantly confined to six hypervariable hairpin loops in the variable domains, called complementarity-determining regions^5^ (CDR) (Figure 1a). TCR chains are assembled somatically during T cell development by the joining of discrete V, (D) and J gene segments by recombination activating gene (RAG)1 and RAG2 (Figures 1b and 2). The process of V (D) J recombination is such that CDR1α, CDR1β, CDR2α and CDR2β are entirely encoded in germline DNA segments. In contrast, the CDR3 loops are the product of junctional diversity and are consequently hypervariable (Figure 1b).

Gene rearrangement is an essential event in the life of a T cell. Expression of RAG1 and RAG2 is lymphoid-specific and dictates irreversible T cell lineage commitment in developing thymocytes.^6^ Moreover, gene rearrangement provides the T cell compartment with sufficient diversity to sustain protective immunity. Indeed, the importance of receptor diversification is apparent in murine models and in a number of primary immunodeficiencies in humans. For instance, *rag*-deficient mice are devoid of T and B lymphocytes.^7^ In humans, *rag* deficiency is linked to severe combined immunodeficiency, and other *rag* mutations can lead to immunodeficiency with expansion of γδ T cells or with idiopathic CD4^+^ T cell lymphopaenia. Mis-sense mutations in *rag1* and *rag2* are the cause of Omenn syndrome, a disease with graft-*versus*-host disease-like clinical presentation.^8,9^ Omenn syndrome is characterized by expansion of autoreactive CD4^+^ T cells with an oligoclonal repertoire and is fatal to infants between 2 and 6 months of age as a result of recurrent infections.^10^

The theoretical diversity in the TCR-αβ repertoire is estimated at 10^15^ clones in mice^11^ and 10^18^ in humans.^12^ Most of these specificities will never be used during an individual's lifetime, as the murine and human peripheral repertoires are composed of 2 million^13^ and 25 million^14^ clonotypes, respectively. Out of 25 million human TCRs, some clonotypes referred to as ‘public' TCRs can be found in different individuals, while others are largely unique to an individual and are said to be ‘private'.

The molecular principles that dictate which TCRs are assembled and selected to seed the peripheral pool, which are shared between individuals and which are private are only starting to emerge. Dissecting the distribution of TCR clonotypes within an individual, and across individuals, in health and disease is critical to our understanding of protective T cell-mediated responses. In this review, we discuss the various factors working to shape the pre-selection, naive and antigen-specific TCR repertoires. We bring particular attention to recent studies which suggest that TCR ‘signatures' shared across genetically disparate individuals may become important diagnostic tools and predictors of immune protection or disease.

## TCR diversity in the pre-selection repertoire

Gene rearrangement is typically thought of as an inherently random process. Intuitively, stochastic diversification of the repertoire would seem advantageous, maximizing potential immune coverage without prior bias towards certain specificities. However, numerous studies have demonstrated that the complexity of the repertoire is not achieved at random. Rather, generation of diversity in αβ T cells is tightly regulated and the composition of the repertoire, even prior to thymic selection is highly structured.

Both genetic and epigenetic factors influence the composition of the pre-selection repertoire. The ‘accessibility hypothesis'^15^ posits that in order for recombination to take place, gene segments must first be made accessible to the recombination machinery. This in turn depends on subnuclear relocation of the rearranging TCR loci (*tr*), DNA methylation status, recruitment of chromatin remodelling enzymes, histone modification and germline transcription. The mechanisms involved in spatial and temporal control of V (D) J recombination have been reviewed elsewhere.^16^

Activation of the 3′ proximal region of antigen receptor loci is well characterized and known to be dependent on activation of a local enhancer. However, the factors that govern the accessibility and activation of the 5′ V region are less clear.^17,18^ In particular, whether differential accessibility and activation status of V genes can affect the composition of the resulting repertoire is largely unknown. In the immunoglobulin heavy chain (*Igh*) locus, distal V genes have been shown to have higher levels of active histone markers compared to proximal segments, which suggests that different V elements recombine at different frequencies despite being equally accessible to the recombination machinery.^19^ Whether this is also true for *tr* loci has yet to be determined.

Recently, a comprehensive analysis of the mouse TCR-α repertoire revealed that the frequency of out-of-frame sequences was dependent on V and J segment usage, suggesting that the production of out-of-frame, non-functional TCR-α chains is genetically determined.^20^ Moreover, syngeneic mice have been shown to display at least 15% overlap in the TCR-β chain repertoire. Most of this overlap is attributed to recombinatorial bias, as the vast majority of shared sequences are observed even in the pre-selection repertoire.^21^ An earlier study looking into the TCR-β chain repertoire of TCR-α^−/−^ mice had already suggested recombinatorial bias in the pre-selection repertoire. In TCR-α^−/−^ mice, the TCR-αβ receptor is not expressed, providing insight into the composition of the rearranged TCR-β pool in the absence of thymic selection. In this setting, 11% of all analysed sequences were repeats.^22^

Altogether, these studies, and others, suggest bias in the composition of the TCR repertoire prior to any contribution from thymic selection. Notably, V (D) J recombination determines the extent of TCR sharing between different individuals, irrespective of MHC haplotype.

## TCR diversity in the naive pool

**Figure 3:**
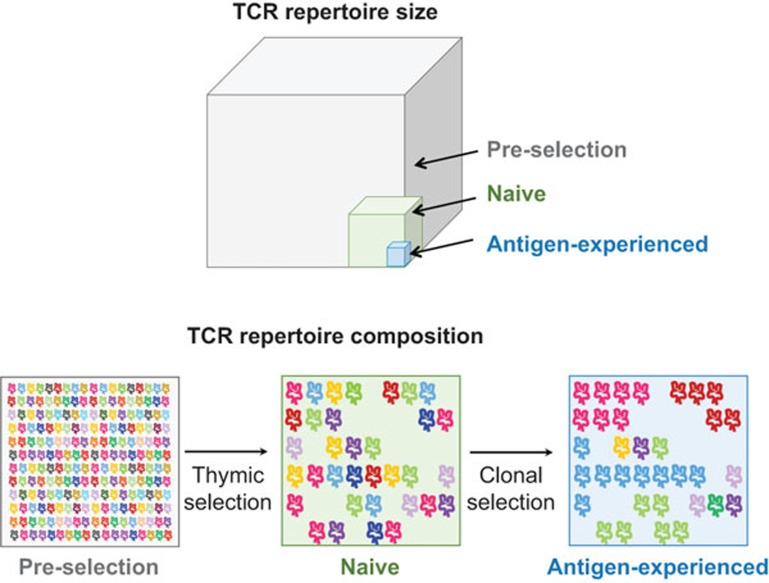
Size and composition of the pre-selection, naive and antigen-experienced repertoires. TCR diversity is greatest in the pre-selection repertoire (gray). Positive and negative selection in the thymus purges the pre-selection repertoire of most specificities, creating a peripheral naive repertoire that is substantially less diverse (green). In the periphery, antigen exposure further narrows the repertoire over time leading to clonal expansion of antigen-specific populations (blue). TCR diversity is largely preserved throughout the human lifespan, except in infancy and old age, but the net distribution of TCR clonotypes is altered. TCR, T cell receptor.

Successfully rearranged TCRs are expressed at the T cell surface and audition for selection on thymic self-pMHC ligands. The net result of thymic selection is that the post-selection repertoire is largely purged of most clonotypes.^23^ Typically, only one in a hundred thymocytes are thought to be granted access to the periphery^24^ (Figure 3). Assessing the relative distribution of TCR clonotypes has long been a challenge in the naive pool because of low precursor frequency. Nevertheless, identifying the factors that shape the composition of the naive repertoire is critical to our understanding of protective T cell-mediated immunity because naive lymphocytes represent the precursor pool from which all immune responses arise.

The size and diversity of the post-selection thymocyte population is regulated by the ligands made available in the thymus by antigen processing and presentation.^25,26^ Polymorphism at the *mhc* will affect an individual's TCR repertoire by determining the collection of peptides that can be presented to T cells during development.^27,28,29^ The role of the MHC-bound peptide in positive selection was made clear from experiments with foetal thymic organ cultures in which the major route for class I MHC loading and surface expression was blocked by deficiency in the transporter associated with antigen processing 1. In transporter associated with antigen processing 1-deficient foetal thymic organ cultures, the addition of exogenous peptide enhanced class I expression and improved the selection of mature CD8^+^ T cells.^30^ Later experiments demonstrated that larger peptide mixtures were better at positively selecting T cells with a broad repertoire than single peptides,^31^ suggesting that peptide diversity in the thymic stroma correlates with TCR diversity.

At least one other component of the antigen processing machinery, ER-associated aminopeptidase 1 (ERAP1) is also highly polymorphic. Single nucleotide polymorphisms in ERAP1 are inherited as haplotypes, some of which are linked to autoimmune disease.^32^ ERAP1 trims peptides entering the ER in order to increase the frequency of peptides of appropriate length for binding to class I MHC molecules. Of note, ERAP1 activity can lead to peptide over-trimming and destruction. Therefore, allelic variants of ERAP1, with distinct trimming properties, will also influence the composition of the repertoire by determining the identity and abundance of peptides presented to CD8^+^ T cells.^32^

Nitta *et al.*^33^ have shown that mice deficient in the thymoproteasome subunit β5t select fewer CD8^+^ T cells than β5t-sufficient mice. In this setting, CD8^+^ T cells display reduced TCR diversity, which demonstrates that the peptides generated by the unique catalytic activity of the thymoproteasome affect the composition of the repertoire. Similarly, in knock-in mice in which the immunoproteasome β5i subunit replaces β5t, positive selection is compromised.^34^

In summary, TCRs auditioning for selection are picked in a deterministic manner. The factors controlling selection encompass some of the major components of the antigen processing machinery, which determine the peptide universe generated in the thymus.

## TCR bias in antigen-specific responses

The thymus involutes with age and thymic output is reduced as a consequence but the composition of the repertoire remains remarkably constant throughout life, except in infancy and old age.^35^ Homeostatic regulation ensures that most specificities generated by the thymus are maintained during the lifetime of the individual.^36,37^ However, the relative abundance of each specificity is modulated by the individual's history of antigen exposure, as antigen-driven selection in the periphery leads to differential expansion of specific TCR clonotypes. It follows that TCR diversity is the highest in the naive compartment, with the antigen-experienced repertoire being skewed towards just some of these specificities^36,38^ (Figure 3).

In mice, the emergence of a diverse repertoire is a predictor of good disease outcome. Messaoudi *et al.*^29^ showed that wildtype C57BL/6 mice (H-2^b^) infected with *herpes simplex virus*-1 select for a significantly narrower repertoire, compared to a co-isogenic strain (H-2^bm8^) which differed only by expression of a H-2K molecule with four amino acid mutations in the peptide-binding groove. Strikingly, H-2^bm8^ mice showed resistance to infection and increased survival compared to their wild-type counterpart, which led the authors to conclude that the expansion of additional TCRs was necessary and sufficient to confer protection against *herpes simplex virus*-1. Indeed, in this system, *herpes simplex virus*-1 resistance was strictly conveyed by Vβ8-expressing T cells.

Some human infections paint a different picture. Infections with common pathogens give rise to highly skewed and predictable repertoires with clonotypes reported to be shared across HLA-matched individuals. Thus, the advantage of having a diverse repertoire is not always apparent in human disease. The occurrence of shared TCRs is explained by convergent recombination, whereby certain TCR sequences are produced at high frequency (this is the case for near germline sequences requiring few nucleotide additions), and by the selection of TCRs with a selective advantage such as structural features that are optimal for pMHC recognition.^39^

In SIV-infected rhesus macaques, the emergence of public Gag-specific clonotypes correlates with protection and the absolute number of shared clonotypes inversely correlates with viral load.^40^ Similarly in humans, control of HIV-1 replication in the absence of antiretroviral therapy is mediated by a few TCR clonotypes which are selected in the context of the so-called ‘protective MHC alleles' HLA-B*57, HLA-B*27 and HLA-B*58.^41^ These findings imply that certain clonotypes shared across several distinct individuals can be reliably associated with protective, beneficial immune responses and may act as surrogate predictors of disease outcome.

Many T cell malignancies are characterized by extreme clonal skewing, with oligoclonal or even monoclonal T cell expansions. Recently, Clemente *et al.*^42^ showed that in HLA-A2^+^ patients with T-large granular lymphocyte leukaemia, Vβ17-expressing T cells share a unique CDR3 sequence. Importantly, sequencing depth in this study was sufficient to establish that this canonical TCR-β sequence associated with T-large granular lymphocyte leukaemia was largely undetectable in healthy individuals. Similarly, another study following the outcome of autologous stem cell transplantation for the treatment of juvenile idiopathic arthritis suggested that TCR-β oligoclonality was linked to clinical relapse.^43^ Two patients in complete remission displayed diverse TCR-β CDR3 immediately following transplant and at later follow-up time points, whereas the third patient presented with oligoclonal CD8^+^ T cell skewing in most Vβ families and relapsed within a month. Moreover, in the latter case, the dominant clones were shown to have emerged both from the pre-transplant pool and from *de novo* TCR rearrangement. Thus, because TCR skewing arises in a predictable fashion following an antigen-specific response, monitoring TCR oligoclonality and tracking specific TCR clonotypes linked to malignancy, or other immunological disorders, may prove beneficial in the clinical setting.

## TCR diversity in health and disease

Inbred mice represent a powerful tool for the analysis of pre-selection, immunologically naive and antigen-specific repertoires. Murine models first suggested that in a normal setting, the formation of the TCR repertoire was H-2-dependent^44^ and that TCR usage was altered in the context of autoimmune disease.^45,46^ For instance, the non-obese diabetic mouse regulatory T cell (T_reg_) repertoire is significantly restricted compared to conventional T cells and to T_reg_ from wild-type mice. Strikingly, this defect in generation of diversity is apparent in *rag*^−/−^ B6 mice reconstituted with non-obese diabetic bone marrow indicating a cell-intrinsic origin, i.e., independent of the nature of the selecting thymic stroma. Furthermore, using different congenic non-obese diabetic strains, it was shown that T_reg_ diversity was in fact regulated by a yet-unidentified gene on chromosome 4.^47^ This suggests that unknown genetic mechanisms may be at work to modulate diversity in the T_reg_ lineage, which in turn is critical to the establishment of tolerance. Similarly in humans, TCR diversity is known to be critical for homeostasis of T_reg_ cells and suppressor function.^48^ Therefore, understanding the mechanisms underlying the formation of the T_reg_ repertoire will further our understanding of autoimmune disease and provide insight into how selection of a dysfunctional repertoire leads to disease.

In humans, twin studies are invaluable tools for dissecting the genetic factors underlying the shaping of the peripheral repertoire. Examples of repertoire analysis in twins, particularly hyperanalytical methods such as high-throughput sequencing, are still scarce in the literature. Nonetheless, a few select reports are already providing key information. The earliest study of TCR-β usage by Gulwani-Akolkar *et al.*^49^ already highlighted the influence of HLA alleles. HLA-identical siblings were found to have the highest degree of similarity in the TCR-β repertoire, whereas HLA-haploidentical or HLA-mismatched siblings were dissimilar. Later, Davey *et al.*^50^ re-examined this finding in seven pairs of monozygotic twins. The highest degree of similarity was found in the youngest pair (aged 2) and all other twins (aged 5–44) had at least one difference in Vβ segment usage. Four pairs of twins in this study had a history of disease affecting either one (discordant) or both individuals (concordant). In discordant pairs, notable differences were seen in CD8^+^ T cell Vβ usage. For instance, one individual suffering from asthma showed a significant increase in Vβ8 usage and loss of Vβ12, compared to their healthy twin. Differences in Vβ expression patterns were also noted in two other pairs of discordant twins affected by Hodgkin's lymphoma and by polycythemia vera. Interestingly, Vβ usage was also found to diverge in twins concordant for systemic lupus erythematosus. The authors therefore suggested that both environmental factors and genetic factors contributed substantially to shaping the TCR repertoire. Thus, at birth, monozygotic twins have near identical repertoires with respect to Vβ usage, but changes associated with an individual's unique history of antigen exposure arise over time.

A longitudinal study following monozygotic twins simultaneously infected with the same HIV-1 strain by intravenous drug abuse suggested random TCR recruitment in various epitope-specific responses. Over the course of the disease and virus evolution, several Pol and Nef epitopes were found to be concordant (shared between the twins), whereas others were unique to either individual. Remarkably, in these individuals, the TCR-β chain repertoires raised against the shared epitopes were exclusively private. Thus, the identical genetic background and the concordant pathways of viral escape did not lead to TCR sharing in this instance and the authors concluded that the recruitment of pathogen-specific TCRs was essentially stochastic.^51^ This finding therefore suggested that an individual's natural history of antigen exposure may be at least as important as their genetic background in determining the composition of the antigen-specific repertoire and that the forces shaping the repertoire are more complex than previously thought.

## TCR clonotypes as markers of disease

Tracking and detecting TCR ‘signatures' associated with specific human infections may prove problematic considering the complexity and diversity of human pathogens. Even in cases where TCR expansions can be reliably and accurately linked to infection, developing diagnostic tools based on TCR clonotyping may be highly unpractical due to rapid onset and progression of disease, particularly in the case of acute infections. Nonetheless, this approach may become an option in the context of autoimmune disease or cancer. Indeed, for such disorders, pathogenesis is usually slow and, at least in the case of autoimmunity, the onset of symptoms and formal diagnosis can take place several years after autoantibodies are first detectable in serum. TCR-based diagnoses might offer several practical advantages, as technical resolution is rapidly increasing with the development of high-throughput ‘next generation' sequencing methodologies.

As discussed above, a number of studies suggest that Vβ usage is fairly predictable, based on the influence of HLA alleles. Nevertheless, understanding how the composition of the TCR repertoire is altered in the disease setting remains a challenging task due to technical limitations on the one hand, but also to the complex functional and genetic nature of the T cell pool on the other hand. Studies of monozygotic twins that are discordant for autoimmune diseases have been particularly enlightening.

Fozza *et al.*^52^ looked at the repertoires of monozygotic twins discordant for type 1 diabetes at the time of the study. In this study, any individual was found to share more TCR-β clonotypes with their twin than with unrelated individuals. However, the extent to which TCR-β repertoires overlapped was unlinked to disease status. This is to be expected because the presence of a given TCR sequence does necessarily implicate a particular T cell clone in disease pathogenesis. Indeed, using CD8^+^ T cell clones derived from a pair of multiple sclerosis (MS)-discordant twins, Somma *et al.*^53^ identified a number of TCR-β implicated in the pathogenesis of MS which could be clonally expanded either from the healthy or the affected twin.

In contrast, Utz *et al.*^54^ showed that bulk T cell cultures harvested from monozygotic twins concordant for MS mobilized the same TCR-α chains following challenge with myelin basic protein, whereas TCR-α chains from discordant twins were dissimilar. Another study has recently demonstrated that the cerebrospinal fluid of MS patients is enriched with public EBV-cross-reactive TCR clonotypes which, again, could serve as potential markers of disease.^55^ Thus, in these examples, similarities in Vα and Vβ usage were largely linked to genetic factors and not critically influenced by nominal antigen. Differential TCR usage in discordant twins could mirror the role of T cell-mediated responses as drivers of autoimmune disease and mapping how the TCR repertoire is altered by various disease settings will be key to the establishment of TCR-based diagnostic methods. The discrepancies in the aforementioned studies highlight the need for more rigorous and more powerful sequencing approaches coupled to the identification of antigen-experienced T cell subpools. Indeed, developments in T cell phenotyping by flow cytometry are also moving apace, making it possible to identify specific TCRs within a given T cell subpopulation. Determining the frequency and distribution of particular TCR sequences among discrete T cell subcompartments might yet prove to be extremely powerful.^56^

**Figure 4:**
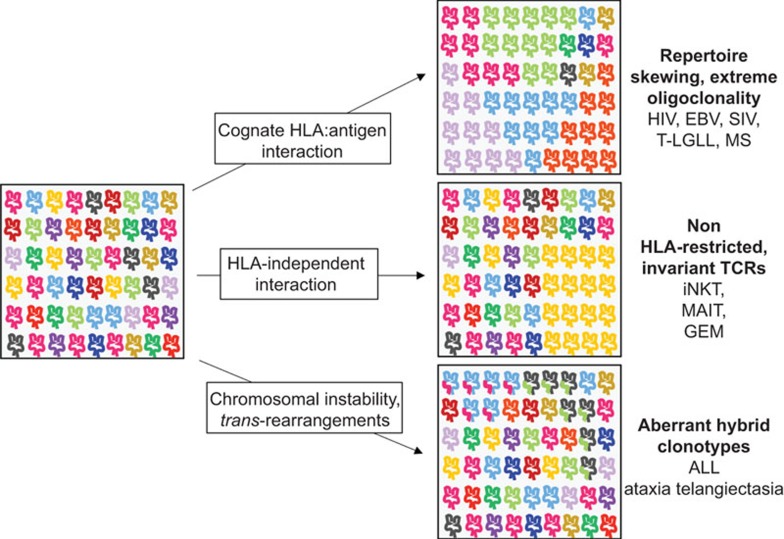
Skewing of the TCR repertoire in human disease. The peripheral TCR repertoire is shaped by antigen encounter and altered in the context of disease. Classical pMHC recognition leads to clonal expansion of antigen-specific T cells, which in some human pathologies can lead to extreme oligoclonality and skewing (top). In this setting, the expansion of public clonotypes can be beneficial as seen in HIV-1 infection, but in other cases, certain clonotypes are involved in disease pathogenesis as described for MS. Other semi-invariant clonotypes such as iNKT (also called NKT type I), MAIT and GEM TCRs expand in response to some microbial infections in an HLA-independent manner (centre). Some malignancies such as ALL, or other disorders associated with chromosomal instability, provoke the expansion of aberrant clonotypes (Ig/TCR hybrids or TCR-γ/TCR-β hybrids; bottom) that are largely absent from the healthy. ALL, acute lymphoid leukaemia; GEM, germline-encoded, mycoyl-reactive; iNKT, invariant natural killer T; MAIT, mucosa-associated invariant T; MS, multiple sclerosis; pMHC, peptide-major histocompatibility complex; TCR, T cell receptor.

Using ‘next-generation' sequencing, a more recent study in three pairs of healthy, monozygotic twins suggested that the composition of the TCR repertoire at the CDR3 level was also largely determined by genetic factors.^57^ V segment usage in out-of-frame sequences was found to be more similar in twins than in non-twins, indicating a genetically determined bias prior to selection, in line with had been previously reported by Genolet *et al.*^20^ Remarkably, monozygotic twins did not share more in-frame sequences with their twin than with an unrelated individual, indicating that certain TCR clonotypes can be enriched and shared across a wide population irrespective of HLA type, as previously suggested by Robins *et al.*^38^ This is in accordance with previous studies highlighting convergent recombination as a major route for the generation of public TCRs. Some of the sequences shared by multiple, unrelated individuals may be enriched with clonotypes derived from invariant populations such as invariant natural killer T cells, mucosa-associated invariant T cells and germline-encoded, mycoyl-reactive T cells.^58^ Such semi-invariant TCRs are likely raised against common antigens and shared across the population. Thus, if linked to certain infections, such TCRs could become invaluable tools for the diagnosis of human disease (Figure 4).

Some diseases are associated with the emergence of aberrant clonotypes. All *tr* loci normally rearrange in *cis*, or in other words, strictly within locus. In pathologies linked to chromosomal instability including various malignancies, *trans* rearrangements arise in the periphery at high frequency. Inversions on chromosome 7 can give rise to Vγ to DβJβ recombination. Such rearrangements represent less than 1 in 100 000 peripheral blood lymphocytes in healthy individuals. However, in patients with ataxia telangiectasia, the abundance of such clonotypes is increased 50- to 100-fold. Similarly, patients with childhood acute lymphoid leukaemia of B-cell lineage present with high frequency of IGHV/Jα hybrids.^59^ In various lymphoma patients, the V (D) J recombination machinery has been implicated in abnormal interchromosomal joining. Thus, *trans* rearrangements are abundant in such patients but either largely absent or extremely rare in healthy individuals and may become important diagnostic markers for several disorders and malignancies (Figure 4).

## Conclusions

T cells orchestrate a variety of immune responses against self- and foreign antigen and the genetic basis of these responses is encrypted in the TCR repertoire. Over the last two decades, numerous studies have accumulated in the literature to suggest that the composition of the TCR repertoire is a tightly regulated quantity. The formation of the pre-selection and the naive repertoires is largely determined by genetic factors, including HLA type and genes encoding the components of the V (D) J recombination and antigen processing machineries. Thus, against a given haplotype, the TCR repertoire is a structured immunological system. However, the statistical distribution of TCR clonotypes incurs large changes over time according to the personal history of antigen encounter. In some cases, even HLA-identical monozygotic twins can raise different TCR pools against the same antigen. Consequently, the TCR repertoire, albeit regulated, and governed by genetic forces, is nonetheless a large and complex system. How the repertoire differs between distinct individuals, whether genetically identical or genetically disparate, is poorly understood and dissecting the composition of the human repertoire in the antigen-experienced compartment has long remained a challenge due to technical limitations. The methodology and the tools required for comprehensive genetic analysis of antigen receptors have only recently begun to unravel the complexity of the T cell compartment. Notably, numerous studies have described how the repertoire might be altered in the context of certain infections, malignancies or immunological disorders. One trending theme is that some pathologies provoke the emergence of specific TCR clonotypes, which may prove to be invaluable immunological ‘signatures' in the clinical setting. Such signatures include public TCR chains raised against common pathogens, extreme oligoclonality and TCR skewing as seen in many lymphoid malignancies, unique clonotypes associated with autoimmune disease or even aberrant TCR hybrid chains that are linked to numerous immunological disorders. These may represent only a few examples of the information encoded in the TCR repertoire and our understanding of the forces governing the complexity of the T cell compartment in health and disease may be key to future diagnostic and therapeutic interventions.
